# A Correlation Analysis of Peripheral Oxygen Saturation and Arterial Oxygen Saturation Among COVID-19 Patients

**DOI:** 10.7759/cureus.24005

**Published:** 2022-04-10

**Authors:** Prashant Sirohiya, Saurabh Vig, Khushboo Pandey, Jitendra K Meena, Ram Singh, Brajesh k Ratre, Balbir Kumar, Anuja Pandit, Sushma Bhatnagar

**Affiliations:** 1 Onco-Anaesthesia and Palliative Medicine, All India Institute of Medical Sciences, New Delhi, IND; 2 Preventive Oncology, All India Institute of Medical Sciences, New Delhi, IND

**Keywords:** arterial blood gas, oxygen saturation, arterial oxygen saturation, peripheral oxygen saturation, coronavirus disease

## Abstract

Background and objective

It has been observed that peripheral oxygen saturation (SpO_2_) measured by pulse oximeter is consistently lower than arterial oxygen saturation (SaO_2_) measured directly by blood gas analysis. In this study, we aimed to evaluate the correlation between SpO_2_ and SaO_2_, and SpO_2_ and partial pressure of oxygen (PaO_2_), and compare the SpO_2_/FiO_2 _(SF) and PaO_2_/FiO_2 _(PF) ratios in patients with coronavirus disease 2019 (COVID-19).

Methods

In this observational study, SpO_2 _was recorded and arterial blood gas analysis was performed among 70 COVID-19 patients presenting on room air (FiO_2 _= 0.21). SaO_2_ and PaO_2_ were recorded from arterial blood gas analysis. The SF and PF ratios were then calculated.

Results

The strength of correlations between SpO_2 _and SaO_2_, and SpO_2_ and PaO_2_, were significant (p<0.001) and moderately positive [Pearson coefficient (r) = 0.68, 0.53]. SpO_2_ value (85%), i.e., SF ratio (404.7 or below), was the best estimate for mild ARDS (acute respiratory distress syndrome) [PF ratio (300 or below)] with a sensitivity of 80.6% and specificity of 53%.

Conclusion

A pulse oximeter is a vital tool in the diagnosis and management of COVID-19. In our study, SpO_2_ was found to have a positive correlation with SaO_2 _and PaO_2 _with acceptable sensitivity but low specificity in estimating mild ARDS. Therefore, pulse oximetry can be used as a tool for the early diagnosis of mild COVID-19 ARDS as per the given considerations and clinical correlation.

## Introduction

Coronavirus disease 2019 (COVID-19), caused by severe acute respiratory syndrome coronavirus 2 (SARS-CoV-2), is an ongoing pandemic, constantly straining the healthcare systems worldwide [1]. Hypoxemic respiratory failure due to viral pneumonia is a common cause of hospital visits and ICU admissions. Further elaboration of the clinical spectrum of SARS-CoV-2 entails the concept of happy hypoxemia, which has been regarded as a clinical condition with no or minimal respiratory distress, despite a low room-air oxygen saturation as shown by a pulse oximeter [typically, peripheral oxygen saturation (SpO_2_) <90%] [2]. The possible pathophysiological mechanism behind this has been hypothesized to be the phenotypic presentation of patients, such as low elastance (and consequent high compliance), low ventilation-perfusion, and low lung recruitment as a consequence of systemic inflammatory response to SARS-CoV-2 infection [3,4].

The expeditious deterioration in the clinical condition leading to acute respiratory distress syndrome (ARDS) has mandated the utilization of other means of oxygen supplementation such as a high-flow nasal cannula, non-invasive ventilation, or endotracheal intubation, depending on the clinical scenario and the judgment of the treating physician [5]. In this context, the necessity for a rapid, non-invasive screening modality for the assessment of peripheral capillary oxygenation and early decision-making for the planning of management is of paramount importance.

The quantification of peripheral oxygen saturation as measured by a pulse oximeter (SpO_2_) has been reported to be a surrogate for partial pressure of oxygen (PaO_2_) for the diagnosis and management of ARDS [6]. It has been observed that oxygen saturation measured by pulse oximeter (SpO_2_) is consistently lower than arterial oxygen saturation (SaO_2_) measured directly by blood gas analysis [7]. However, the literature on the correlation between SpO_2_, SaO_2_, and PaO_2_ in viral pneumonia caused by SARS-CoV-2 is sparse. In light of this, we planned this study to evaluate the correlation between SpO_2_ and SaO_2_, and SpO_2_ and PaO_2_, and compare the SpO_2_/FiO_2 _(SF) and PaO_2_/FiO_2 _(PF) ratios in patients with COVID-19.

## Materials and methods

In this cross-sectional study, 70 consecutive COVID-19 patients who were admitted to a designated tertiary level COVID-19 facility based at the National Cancer Institute (Jhajjar), AIIMS, New Delhi from June 17 to June 30, 2021, were enrolled. The study was approved by the Institutional Ethics Committee (IEC-74/05.02.2021, RP-18/2021). All patients enrolled presented on room air to the hospital. When a patient got admitted to the ward, as a routine practice, the SpO_2_ was recorded by a pulse oximeter (CARESCAPETM Monitor B850, GE Healthcare Finland Oy, Helsinki, Finland with Nellcor™ SpO_2_ technology, Medtronic, Dublin, Ireland), and the arterial blood gas sample was taken in a 2-ml syringe (heparinized) and was sent for arterial blood gas sampling (Blood Gas System GASTAT-700Model, Techno Medica Co., Ltd., Yokohama, Japan). Thereafter, oxygen therapy was initiated based on the assessment of SpO_2_ and clinical signs. SaO_2_ and PaO_2_ were documented from arterial blood gas analysis. The SF ratio and PF ratio were calculated and correlated. FiO_2_ level was taken as 0.21 in all patients.

Adult patients on room air with COVID-19-positive reports either by reverse transcription-polymerase chain reaction (RT-PCR) or rapid antigen test of the nasopharyngeal swab were included in this study. Exclusion criteria were patient refusal to participate in the study, patients showing signs or a history of peripheral ischemia, weak pulses, those with negative modified Allen’s test, respiratory distress (respiratory rate >24/minute), and those experiencing shortness of breath, patients undergoing any sort of oxygen therapy, and those with a systolic blood pressure <80 mmHg; in case of any exclusions or refusals, the next eligible patients were included in the study [8,9].

Sample size calculation

The sample size for the study was calculated by using the Bland-Altman method with data assumptions based on a similar study by Wilson-Baig et al [7]. Assuming 95% confidence interval, 80% power, a mean difference of 5.3%, precision of 1.8%, and a maximum allowed difference of 10%, a minimum sample size of 60 patients was determined using MedCalc V.19.

Statistical analysis

Statistical analyses were performed using SPSS Statistics version 22.0 (IBM, Armonk, NY). Quantitative data were presented as mean ± standard deviation (SD), while categorical data were demonstrated as frequency and proportions (%). The categorical parameters were compared by chi-squared tests, and the continuous variables were compared by independent t-test. The relationship between SF and PF ratios was described by a linear regression equation. Receiver operating characteristic (ROC) curves were plotted to determine the sensitivity and specificity of the SF ratio threshold values correlating with a PF ratio of 300 or below (mild ARDS). A p-value <0.05 was considered statistically significant. A Bland-Altman analysis was performed for the mean difference and proportional bias between SpO_2_ and SaO_2_ and SF and PF ratios.

## Results

Out of the 80 patients originally recruited for the study, 10 patients were excluded. Four patients had poor plethysmogram, four had severe shortness of breath, and two patients had systolic blood pressure <80 mmHg. Of the 70 patients enrolled in this study, most were men (44, 62.9%), and 29 (41.4%) were aged 60 years and above. Based on their body mass index (BMI), most (40, 57.1%) patients were of normal weight while seven (10.0%) were obese. The mean age of the study population was 55.31 ± 14.48 years. The mean weight of the patients was 66.97 ± 8.9 Kg. Table 1 demonstrates the baseline findings of the patients enrolled in the study.

**Table 1:** Baseline clinical characteristics of patients enrolled in the study BMI: body mass index; SD: standard deviation: SF: SpO_2_/FiO_2_; PF: PaO_2_/FiO_2_

Clinical characteristics	Mean ± SD	Range
Age (years)	55.31 ± 14.48	18-88
Weight (kg)	66.97 ± 8.90	48-92
Height (meters)	1.64 ± 0.08	1.45-1.85
BMI (kg/m^2^)	24.90 ± 3.83	15.15-36.79
SpO_2 _(%)	87.07 ± 11.30	55-100
SaO_2 _(%)	87.12 ± 8.92	68.3-99.2
PaO_2 _(mmHg)	66.98 ± 15.94	41.0-121.0
PaCO_2_ (mmHg)	34.19 ± 6.38	22.0-57.1
SF ratio	414.6 ± 53.8	262-476
PF ratio	318.9 ± 75.9	195-576

**Figure 1:**
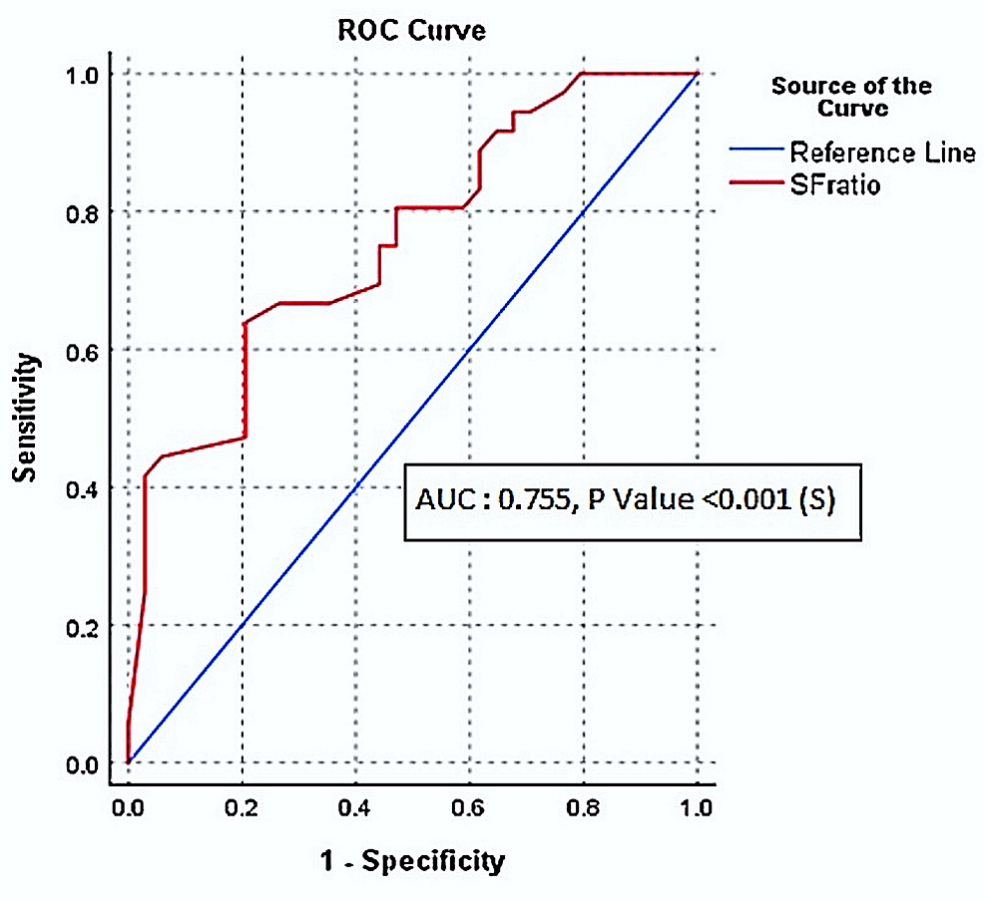
ROC curve analysis for SF ratio values for PF ratio values ≤300 (mild ARDS) [AUC: 0.755, p<0.001 (S)] ROC: receiver operating characteristic; SF: SpO_2_/FiO_2_; PF: PaO_2_/FiO_2_; ARDS: acute respiratory distress syndrome; AUC: area under the curve

Parameters like gender, obesity (BMI), and comorbidity status were found to have no significant relationship with either SF ratio (p = 0.250, 0.340, 0.766 respectively) or PF ratio (p = 0.187, 0.237, 0.813 respectively). However, age had a significant inverse association with the PF ratio (p = 0.048) but not with the SF ratio (p = 0.194). On ROC analysis, an SF ratio cutoff of 404.7 was estimated for diagnosing mild ARDS (PF ratio ≤300) with a sensitivity of 80.6% and specificity of 53% (Figure 1).

**Figure 2:**
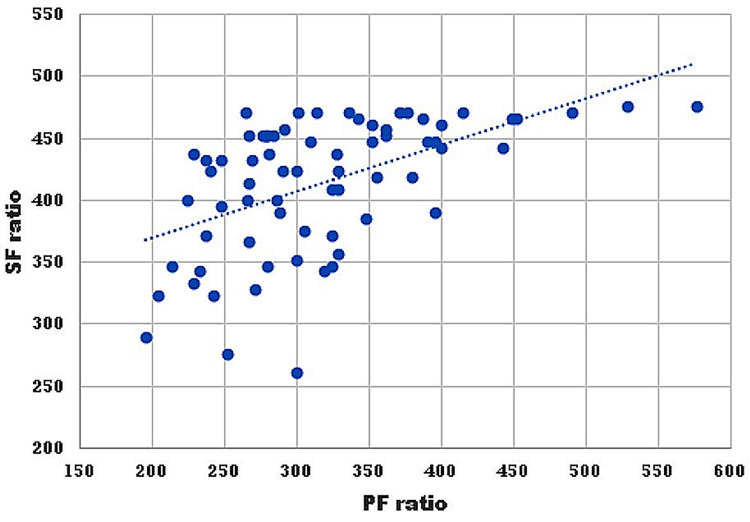
Scatter diagram showing a correlation between SF ratio vs. PF ratio The strength of the correlation is moderately positive [Pearson coefficient (r) = 0.53, p<0.001] SF: SpO_2_/FiO_2_; PF: PaO_2_/FiO_2_

**Figure 3:**
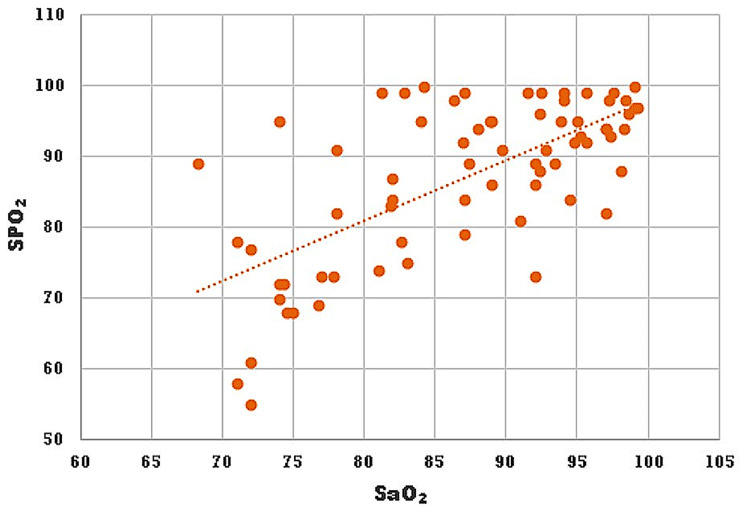
Scatter diagram showing a correlation between SpO2 vs. SaO2 The strength of the correlation is moderately positive [Pearson coefficient (r) = 0.68, p<0.001]

The relationship between SF and PF ratio can be described by the following regression equation: PF ratio = 7.90 + 0.75SF ratio (p<0.001). The strength of correlations between SpO_2_ and PaO_2_ (and SF and PF ratios) [Pearson coefficient (r) = 0.53, p<0.001], and SpO_2_ and SaO_2_ (r = 0.68, p<0.001) were significant and moderately positive (Figures 2, 3).

**Figure 4:**
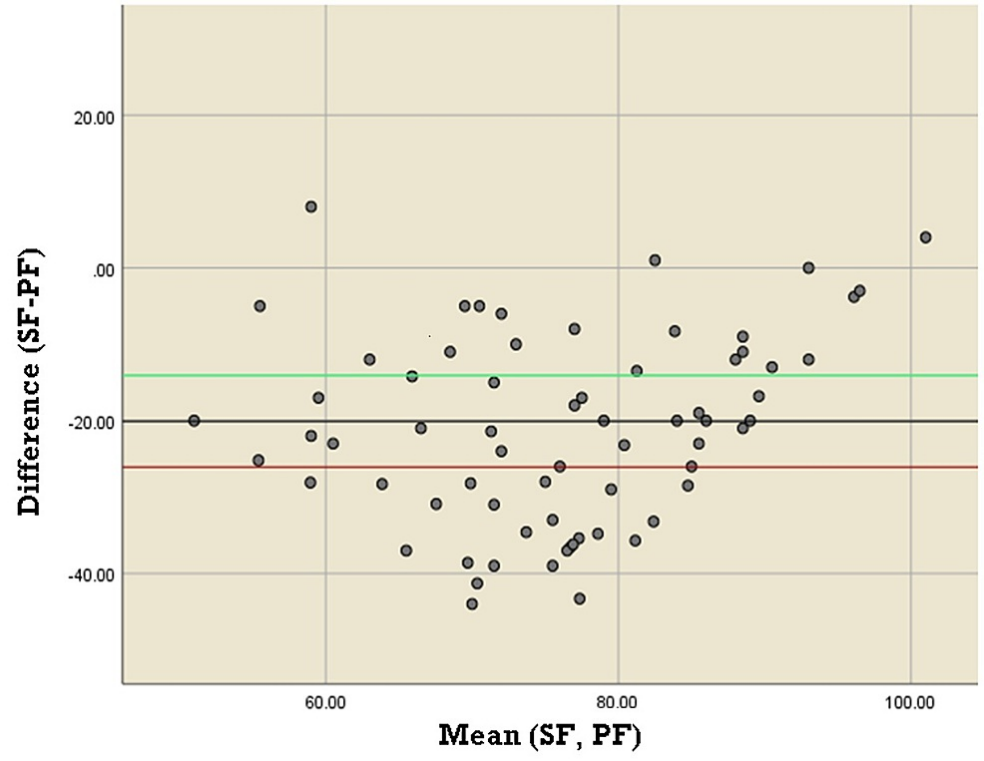
Bland-Altman plot comparing agreement between SF and PF ratio SF: SpO_2_/FiO_2_; PF: PaO_2_/FiO_2_

**Figure 5:**
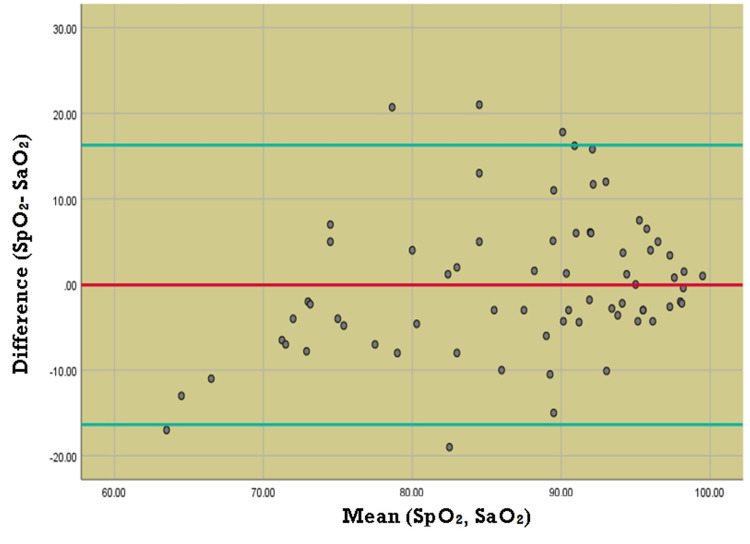
Bland-Altman plot comparing agreement between SpO2 and SaO2

The Bland-Altman plot showed a mean difference of -20.08% between SF and PF ratio measurements with a significant proportional bias (p = 0.001). The Bland-Altman plot between SpO_2_ and SaO_2_ showed a mean difference of -0.048% with a significant proportional bias (p = 0.011). (Figures 4, 5).

## Discussion

The management of COVID-19 patients with respiratory distress mandates utmost vigilance. The severity of signs and symptoms of COVID-19 may range from none or mild to acute respiratory failure requiring immediate intervention [9]. Nevertheless, mild ARDS has often been found to be present in mildly symptomatic and spontaneously breathing patients [10]. The overwhelmed healthcare systems in this COVID-19 era require a greater proportion of quick and less invasive modalities for early screening and management of patients presenting with respiratory distress [11]. One such modality is pulse oximetry, by which SpO_2_ can be recorded. The accuracy of the finger-probe pulse oximeter has been quoted as ±2% at a given range of oxyhemoglobin saturation (70-99%). In patients who are critically ill, SpO_2_ does not reliably indicate SaO_2_ [12,13]. Racial bias can also affect the accuracy of pulse oximetry, with darkly pigmented people at an increased risk of hypoxemia [14].

A study involving 15 patients by Wilson-Baig et al. indicates that SpO_2_ does not reliably predict SaO_2_, with SpO_2_ underestimating SaO_2_ constantly [7]. In their study, SpO_2_ values consistently read lower than SaO_2_, with an average of 5.3% with 95% limits of agreement on Bland-Altman analysis. They have proposed that silent hypoxia in COVID-19 patients at an earlier stage could be explained in part by their observations. Their results may be attributed to the low sample size in their study. In our study of 70 patients, we found that SpO_2_ and SaO_2_ measurements had a minimal mean difference of 0.048% with a significant proportional bias (p = 0.011). On correlational analysis, we found a moderately positive correlation between SpO_2_ and SaO_2_ (r = 0.68, p<0.001), and SpO_2_ and PaO_2 _(r = 0.53, p<0.001).

In our study, we excluded all patients who had any cause of underestimation of SpO_2_ and included only those who had good quality pulse oximetry traces. In 70 patients, PaO_2_ and SpO_2_ were measured with the same FiO_2_, and SF and PF ratios were calculated. The SF ratio cutoff of 404.7 for diagnosing mild ARDS (PF ratio ≤ 300) has a sensitivity of 80.6% and a specificity of 53%. The cutoff accurately estimated (80.6%) of cases with mild ARDS although low specificity (53%) suggests that 47% of individuals who did not have mild ARDS might be misclassified as such (false positives), which can result in overdiagnosis. However, while screen testing and triaging patients with severe diseases, i.e., COVID-19, high sensitivity of a test is important to not miss patients (false negatives). The strength of correlation between SF ratio and PF ratio and that between SpO_2_ and SaO_2_ was moderately positive.

Pulse oximetry has been identified as an important tool for risk stratification and assessment of disease severity in COVID-19. A study by Sinha et al. utilized pulse oximetry as a predictor of the need for intubation by using the respiratory rate-oxygenation (ROX) index, which is defined as the ratio of SF to respiratory rate in breaths per minute [15]. Lipworth et al. have utilized the SF ratio along with clinical biomarkers and concluded that rising biomarkers in conjunction with falling SF ratio or PF ratios have serious potential implications in the management of COVID-19 patients. Several studies have incorporated the SF ratio and PF ratio for computing diagnostic and management strategies in ARDS [16-18].

We performed a cross-sectional study among COVID-19 patients to determine the relationship between SaO_2_ and SpO_2_ and compared the SF ratio and PF ratio and found that they correlate to each other in COVID-19 patients; however, the trend is inconsistent as the Bland-Altman plot showed a mean difference of -20.08% between SF and PF ratio measurements with a significant proportional bias (p = 0.001).

This study has a few limitations. We included patients who presented on room air only. We did not include patients who were having unoptimized comorbidities. We used the same brand of the pulse oximeter and ABG machine in our study to reduce bias. Hence, our results may differ from those of studies that use any other pulse oximeter and ABG machine. Also, we did not assess the role of skin pigmentation in pulse oximetry in our study. Future studies should focus on patients with moderate to severe COVID-19 ARDS to further establish the correlation between SpO_2_ and SaO_2_.

## Conclusions

SpO_2_ was found to have a positive correlation with SaO_2_ and PaO_2_. The SF ratio has acceptable sensitivity but low specificity in estimating mild ARDS. Therefore, pulse oximetry can be used as a tool for the early diagnosis of mild COVID-19 ARDS as per the given considerations and clinical correlation.
